# An eco-friendly separation-based framework for quantitative determination and purity testing of an antihypertensive ternary pharmaceutical formulation

**DOI:** 10.1186/s13065-023-00926-1

**Published:** 2023-03-10

**Authors:** Hoda M. Marzouk, Nada S. Ayish, Badr A. El-Zeany, Ahmed S. Fayed

**Affiliations:** Analytical Chemistry Department, Faculty of Pharmacy, Cairo University, Kasr El-Aini St., Cairo, 11562 Egypt

**Keywords:** HPTLC-densitometry, Capillary zone electrophoresis (CZE), Amiloride hydrochloride, Hydrochlorothiazide, Timolol maleate, Hydrochlorothiazide related impurities

## Abstract

Designing new, verified methodologies with a focus on sustainability, analytical efficiency, simplicity, and the environment has become a major priority for pharmaceutical quality control units. In this way, sustainable and selective separation-based methodologies were designed and validated for the concurrent estimation of amiloride hydrochloride (AML), hydrochlorothiazide (HCT) and timolol maleate (TIM) in their fixed dose formulation (Moducren^®^ Tablets) along with hydrochlorothiazide potential impurities, salamide (DSA) and chlorothiazide (CT). The first method is a high performance thin layer chromatographic method (HPTLC-densitometry). The first developed method employed silica gel HPTLC F_254_ plates as stationary phase using a chromatographic developing system composed of ethyl acetate–ethanol–water–ammonia (8.5:1:0.5:0.3, by volume). The separated drug bands were densito-metrically measured at 220.0 nm for AML, HCT, DSA and CT and at 295.0 nm for TIM. The linearity was assessed over a wide concentration range, 0.5–10 µg/band, 1.0–16.0 µg/band and 1.0–14 µg/band for AML, HCT and TIM, in order and 0.05–1.0 µg/band for each of DSA and CT. The second method is capillary zone electrophoresis (CZE). The electrophoretic separation was achieved using background electrolyte (BGE), borate buffer 40.0 mM with pH 9.0 ± 0.2, at applied voltage of + 15 kV with on-column diode array detection at 200.0 nm. The method linearity was reached over the concentration range of 20.0–160.0 µg/mL, 10.0–200.0 µg/mL, 10.0–120.0 µg/mL for AML, HCT and TIM, respectively and 10.0–100.0 µg/mL for DSA. The suggested methods were optimized to achieve best performance and validated agreeing with the ICH guidelines. Assessment of methods’ sustainability and greenness was performed using different greenness assessment tools.

**Graphical Abstract:**

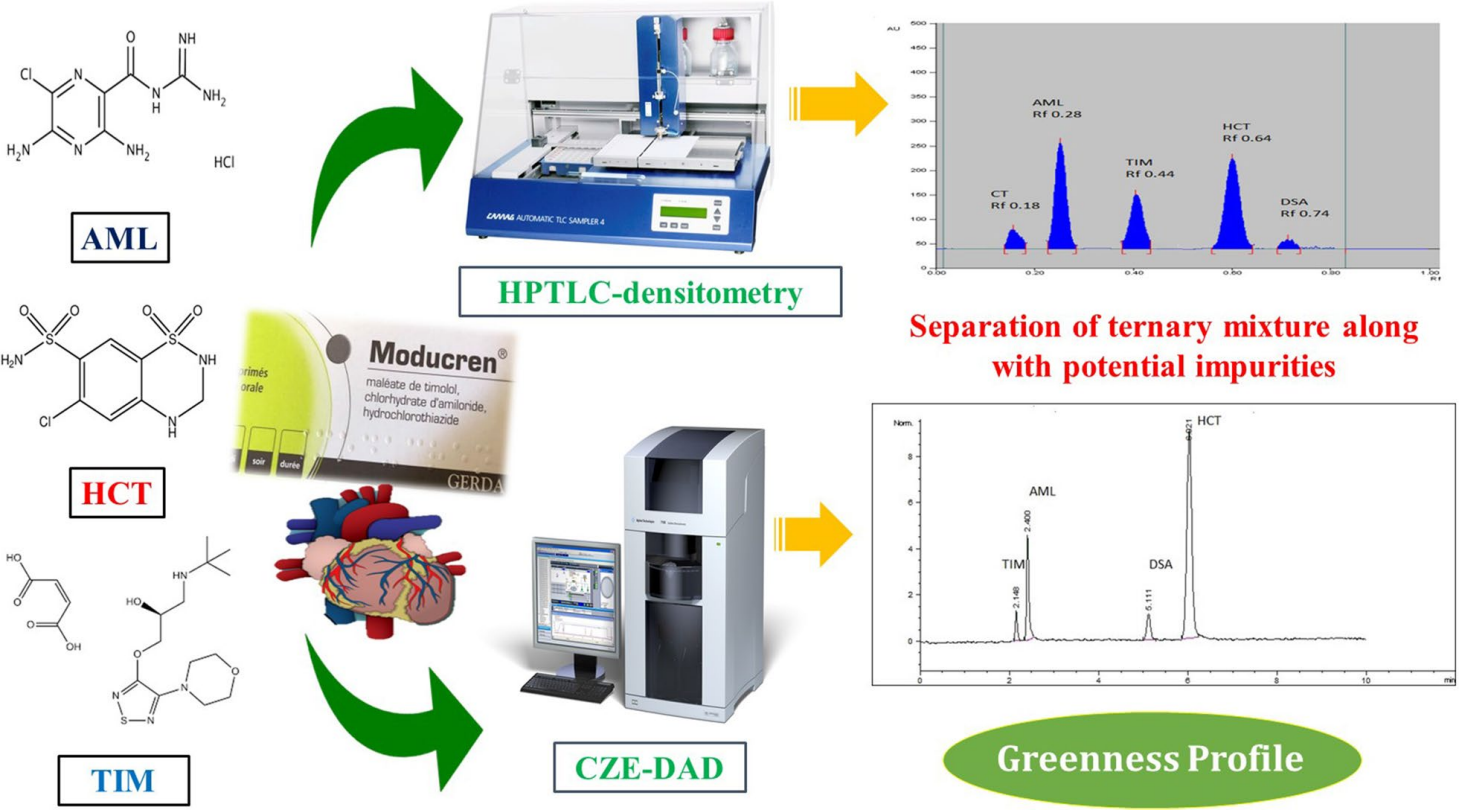

**Supplementary Information:**

The online version contains supplementary material available at 10.1186/s13065-023-00926-1.

## Introduction

Due to the persistent contamination of the ecosystem and the surrounding atmosphere with different types of pollutants, the whole world recently suffers from climatic changes that has a potential impact on all living organisms. That is why environmental sustainability became an international concern in order to secure a healthy environment for the next generations to live in [1]. Lately, scientists all over the world are trying to stick to Green Analytical Chemistry concepts with the aim to eliminate or at least minimize the amount of hazardous chemicals used and generated daily to minimize the environmental impacts of the analytical methods [2]. For this reason different metrics were developed to assess the developed analytical method greenness and evaluate its environmental impact [3].

Diverse analytical approaches have been presented to resolve mixtures of multi-component pharmaceuticals as a result of the broad use of separation-based techniques that depend on the point of differential migration. High-performance thin layer chromatography (HPTLC) has grown significantly as an analytical technique, thanks to the introduction of modern instruments, enhanced stationary phases and automated procedures. It has been used widely in pharmaceutical industry for process development and quality control of the active pharmaceutical ingredients (API) and the final drug products. This can be credited to its simplicity, speed, sensitivity, and affordability [1, 4]. On the other hand, capillary zone electrophoresis (CZE) is considered to be a powerful analytical technique that can be used effectively for the analysis of many pharmaceutical and biopharmaceutical compounds [5, 6]. It is also known for its excellent separation efficiency using a very small sample volume with consumption of small amount of aqueous buffers in a short run time. Accordingly, it is considered to be a superior green, environmentally-benign alternative to many conventional chromatographic methods [7, 8]. Furthermore, coupling of DAD detection with CZE permits the analysis at multiple wavelengths at a time, and thereby increase the sensitivity [9], also it helps to identify peaks according to their UV–Visible spectra with peak purity assessment [10].

**Fig. 1:**
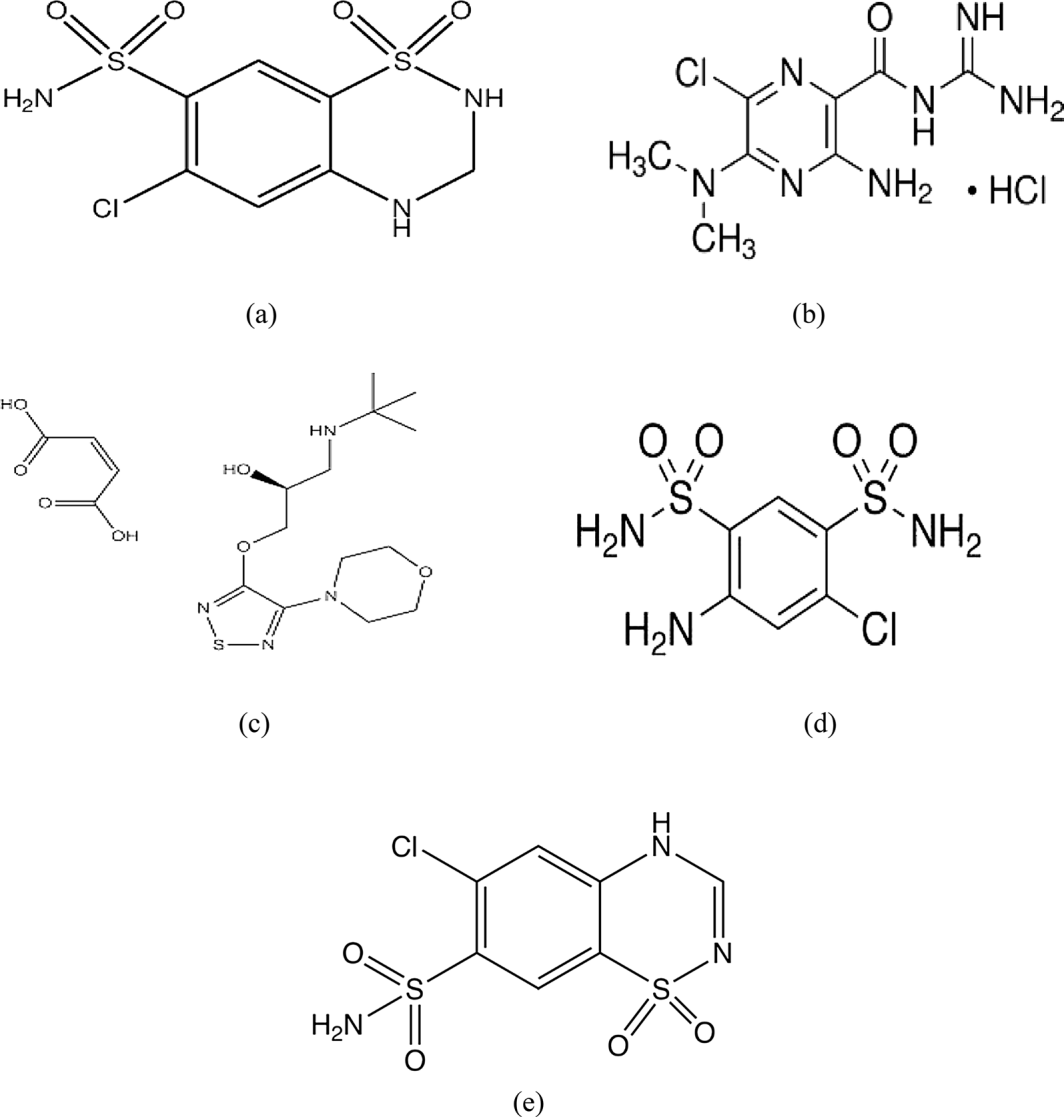
Chemical structure of **a** Hydrochlorothiazide (HCT), **b** amiloride hydrochloride (AML), **c** timolol maleate (TIM), **d** salamide (DSA) and **e** chlorothiazide (CT)

Hypertension is one of the main risk factors for almost all cardiovascular disorders [11]. That is to say good control of blood pressure is a must and it is the main goal of all healthcare providers. Despite the availability of contemporary, efficient antihypertensive medications, the majority of patients continue to have inadequate blood pressure control. To achieve the therapeutic objectives, the majority of hypertension patients will require a combination of antihypertensive medications [12]. Hydrochlorothiazide (HCT), Fig. 1a, 6-chloro-3,4-dihydro-2H-1,2,4-benzothiadiazine-7sulfonamide 1,1-dioxide [13], it belongs to benzothiadiazine class of diuretics. It is an extensively utilized thiazide diuretic that combines with several antihypertensive drugs for effective treatment of hypertension [14]. Amiloride hydrochloride (AML), Fig. 1b, is 3,5-diamino-6-chloro-*N*-(diaminomethylidene) pyrazine-2-carboxamide;hydrochloride [13]. It is a potassium sparing diuretic commonly used in the treatment of hypertension in order to preserve normal serum potassium level [15]. Timolol maleate (TIM), Fig. 1c, with chemical name (2*S*)-1-[(1,1-Dimethylethyl)amino]-3-[[4-(morpholin-4-yl)-1,2,5-thiadiazol-3-yl]oxy]propan-2-ol (*Z*)-butenedioate [16], is a non-selective blocker of the beta-adrenergic receptor utilized effectively in the management of elevated blood pressure especially in patients with mild to moderate hypertension. It works by blocking β_2_ receptors in the blood vessels decreasing the peripheral vascular resistance leading to a decrease in the blood pressure [17]. The co-formulation of the three drugs together was found to have an enhanced antihypertensive effect [12].

It is known that hydrochlorothiazide purity is a challenging problem for the pharmaceutical industry [18, 19]. Salamide (DSA), Fig. 1d, 4-amino-6-chlorobenzene-1,3-disulphonamide and chlorothiazide (CT), Fig. 1e, 6-chloro-2H-1,2,4-benzothiadiazine-7-sulphonamide-1,1-dioxide are stated as hydrochlorothiazide official impurities with specified pharmacopeial limit [16]. It was reported that DSA is considered to be the main degradation product obtained upon hydrolysis of HCT [20], while CT is believed to have less diuretic activity compared to the parent drug [21].

Upon surveying the literature, no method is designated for the determination of the cited antihypertensive drugs simultaneously in their commercially available dosage form or even their determination along with some of their potential impurities. Only few methods were reported recently for the determination of the studied drugs either in their synthetic mixtures or laboratory prepared dosage form, including chromatographic method [22] and spectrophotometric method [23]. Additionally a chemometric method for the determination of hydrochlorothiazide, amiloride hydrochloride and timolol maleate in addition to atenolol has been published [24].

This method endeavors to establish new eco-friendly HPTLC-densitometry and capillary zone electrophoresis methods for the concurrent determination of AML, HCT and TIM in their commercially available pharmaceutical formulation and in the presence of DSA and CT, replacing the common hazards of conventional HPLC methods. Additionally, assessment of the proposed methods greenness using commonly and recently introduced evaluation tools; Eco-scale, NEMI, GAPI and AGREE.

## Experimental

### Reagents and materials

HPLC grade solvents were used, and water was double distilled. Orthophosphoric acid, hydrochloric acid, sodium tetraborate decahydrate and ammonia solution (25%) were all obtained from Sigma Aldrich (Steinheim, Germany). Ethyl acetate and ethanol were purchased from Fisher scientific (UK).

Pure amiloride hydrochloride, with purity of 99.81 ± 1.51%, according to the official method [13], was purchased from Sigma Pharmaceuticals Industries (El Monofeya, Egypt). Pure hydrochlorothiazide was obtained from October Pharma (Cairo, Egypt) and according to the official method [13], its purity was found to be 100.24 ± 1.42%. Timolol Maleate was purchased from Orchidia Pharmaceutical Industries (Obour City, Egypt) with 99.93 ± 1.12% purity according to the official method [16]. Hydrochlorothiazide impurities; salamide and chlorthiazide, were acquired from Sigma Aldrich Chemie (Steinheim, Germany). According to supplier certificate of analysis, their purities were reported to be 99.70 and 99.60%, respectively. Moducren^®^ Tablets were manufactured by GERDA (Paris, France), Batch No. AJC 066, with labelled claim of 2.5 mg AML, 25 mg HCT and 10 mg TIM per tablet and were purchased from the French local market.

### Instrumentation and separation conditions

#### For HPTLC-densitometric method

Samples were spotted as separate bands on 20 × 10 cm aluminum sheets pre-coated with silica gel 60 F_254_ (Merck, Darmstadt, Germany), using sample applicator, Linomat 5 (CAMAG, Muttenz, Switzerland) supplied with a 100.0 µL microsyringe. Bands are separated from one other and from the top and bottom-end of the plate by10 mm with band width of 6.0 mm. Using a CAMAG glass chamber previously saturated with 60 mL mobile phase composed of ethyl acetate–ethanol–water–ammonia (8.5:1:0.5:0.3, by volume), a Linear ascending development was performed over a distance of 8 cm then removed from the chamber and left to dry at room temperature. The separated bands were scanned using a CAMAG TLC scanner at 220.0 nm for AML, HCT, DSA and CT and 295.0 nm for TIM. Scanning was performed using reflectance-absorbance mode of measurement with deuterium lamp as a radiation source and slit dimension kept at 3 × 0.45 mm at scanning speed of 20 mm/s. WinCATS^®^ software was used for densitometric evaluation and the output consist of a densito-grams and integrated peak area.

#### For CZE-DAD method

Using an Agilent 7100 CE instrument supplied with an autosampler, and a diode array detector, electrophoretic separations were performed. The management of the CE system, data collection, and inspection were done using the ChemStation software (Agilent Technologies, Germany). Fused silica capillary, purchased from Agilent Technologies (USA), of total length 48.5 cm, 40 cm effective length and internal diameter of 50 µm was utilized. Separation was achieved using optimized background electrolyte (BGE) of 40.0 mM borate buffer at pH = 9.0, applied voltage of + 15 kV and detection wavelength of 200.0 nm. Each run pass through three main steps; first step is pre-conditioning where 0.1 mol/L NaOH was flushed for 1 min followed by deionized water and background electrolyte (BEG), in order, for 3 min each. Second step is sample injection where sample is injected hydro-dynamically at 50 mbar for 5 s. Finally, the capillary is flushed with deionized water for 5 min as post-run step. During working day, the capillary was flushed with running buffer for 120 s between each two sequential injections. To preserve the repeatability of the injections from run to run, buffer vials were replenished after every five subsequent runs.

### Standard solutions

#### For HPTLC-densitometric method

Standard stock solutions of AML, HCT and TIM (1.0 mg/mL) were prepared by weighing and dissolving 50 mg of each in three separate 50-mL measuring flask, and making the volume to mark using methanol as a solvent.

Stock standard solution of DSA and CT (100.0 µg/mL) were prepared, separately, by weighing 10 mg of each into 100-mL measuring flask and completing the volume using methanol.

#### For CZE-DAD method

Stock standard solutions of AML, HCT, TIM (1.0 mg/mL) and DSA (100.0 µg/mL), each was prepared separately as mentioned above using ethanol as solvent.

### Procedures

#### Construction of calibration curve

##### For HPTLC-densitometric method

Into a set of 10-mL measuring flasks, serial dilutions of the studied drugs were separately performed using methanol as a solvent to prepare working solutions covering the concentration range of 50.0–1000.0 µg/mL for AML, 100.0–1600.0 µg/mL for HCT, 100.0–1400.0 µg/mL for TIM and 5.0–100.0 µg/mL of each of DSA and CT. A volume of 10 µL of respective concentration was spotted, in triplicate, as separate bands and mentioned chromatographic conditions was followed as mentioned. Calibration curves were constructed relating the average obtained peak areas to the corresponding concentrations and then regression equations were computed.

##### For CZE-DAD method

Aliquots of stock standard solutions AML, HCT, TIM and DSA were, separately, transferred into a series of 10-mL measuring flask and the volume was made up to the mark using water as a solvent to cover a concentration range of 20.0–160.0 µg/mL for AML, 10.0–200.0 µg/mL for HCT, 10.0–120.0 µg/mL for TIM and 10.0–100.0 µg/mL for DSA. Samples were injected under hydrodynamic mode. Calibration curves were plotted relating the resulting corrected peak areas to the corresponding concentrations of each component and regression equations were then determined.

#### Assay of pharmaceutical formulation

Ten tablets were weighed separately, powdered and uniformly mixed.

##### For HPTLC-densitometric method

An equivalent amount of two tablets was accurately weighted and transferred to 50-mL measuring flask. A volume of 25 mL methanol was further added and then sonicated for 15 min. The flask was finalized to the mark using methanol and then filtered to get a concentration of 100.0 µg/mL for AML, 1000.0 µg/mL for HCT and 400.0 µg/mL for TIM. A 10 µL aliquot equivalent to 1.0 µg/band AML, (10.0 µg/band) HCT and (4.0 µg/band) TIM were spotted, and chromatographic conditions were followed.

##### For CZE-DAD method

An average weight of two tablets was transferred into 50-mL measuring flask using 25 mL ethanol, and the sample prepared solution was then sonicated (15 min). The volume was brought to the mark with the same solvent and subsequently filtered to obtain a concentration of 100.0 µg/mL for AML, 1000.0 µg/mL for HCT and 400.0 µg/mL for TIM. Appropriate aliquots from the sample prepared solution were then transferred into 10-mL measuring flasks and the volume was made up to the mark using water to obtain a concentration within the linearity range of each drug.

Additionally, standard addition technique was done to assess methods’ accuracy.

## Results and discussion

Separation-based techniques are well-established and irreplaceable techniques in worldwide quality control laboratories for routine drug assay. The literature survey revealed the lack of a reported analytical method for the simultaneous determination and purity testing of the studied drugs in their newly introduced pharmaceutical formulation. The presented contribution is devoted to spot the light on the analytical performance and opportunities existing for HPTLC-Densitometry and CZE-DAD (for the first time) in cited drugs' analysis and impurity profiling purposes.

### Optimization of separation conditions

#### For HPTLC-densitometric method

**Fig. 2:**
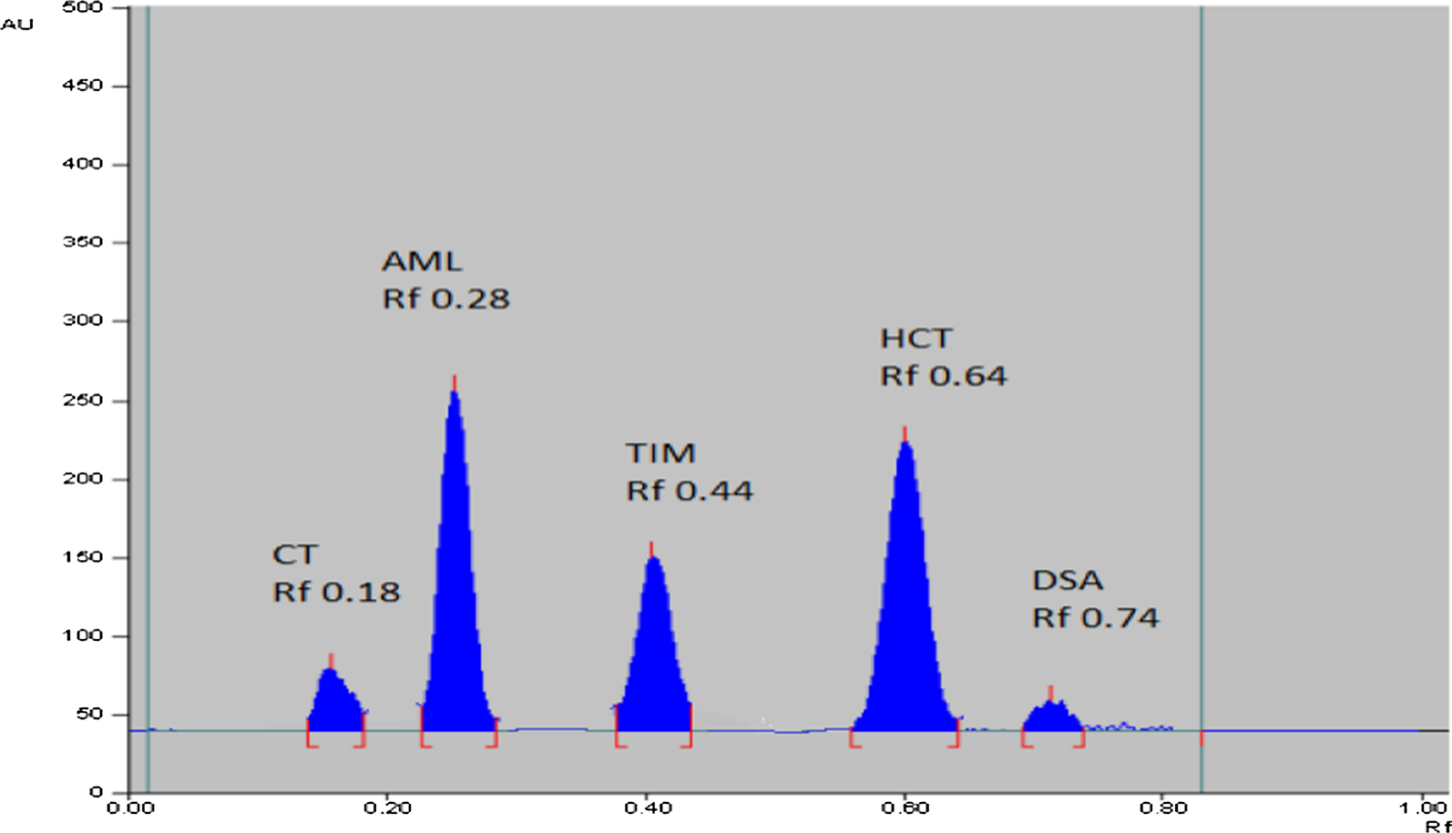
HPTLC-densitogram of mixture of AML (1.0 µg/band), TIM (1.0 µg/band), HCT (1.0 µg/band), DSA (0.05 µg/band), CT (0.05 µg/band) using ethyl acetate–ethanol–water–ammonia (8.5:1.0:0.5:0.3, by volume) as a developing system at 220.0 nm

Minimizing the environmental impact while maintaining method efficiency was one of the key issues faced throughout method development. For the separation of the aforementioned drugs together with HCT impurities, a number of development systems with various compositions and ratios were tested avoiding the most harmful solvents, such as benzene and toluene. Among the tried systems: ethyl acetate–methanol–ammonia with different composition ratios. Worthy separation was achieved between AML and the rest of the drugs, but no separation was observed between the two impurities DSA and CT, with bad resolution of HCT and TIM bands. Trying to replace ammonia with glacial acetic acid, AML peak was observed very close to baseline with bad peak symmetry. Other systems consisting of ethyl acetate–ethanol–ammonia and ethyl acetate–ethanol–water–ammonia in different ratios were tried. It was observed that good resolution and enhanced peak symmetry of the drug mixture and impurities was obtained upon using system consisting of ethyl acetate–ethanol–water–ammonia (8.5:1:0.5:0.3, by volume) as shown in Fig. 2. For densitometric measurement, various scanning wavelengths were tried for optimum quantification including (220.0, 270.0 and 295.0 nm), where 220.0 nm was selected for AML, HCT, DSA and CT determination and 295.0 nm for TIM. These two selected wavelengths found to have the highest sensitivity for the determination of the cited analytes and minimum baseline noise.

#### For CZE-DAD method

**Fig. 3:**
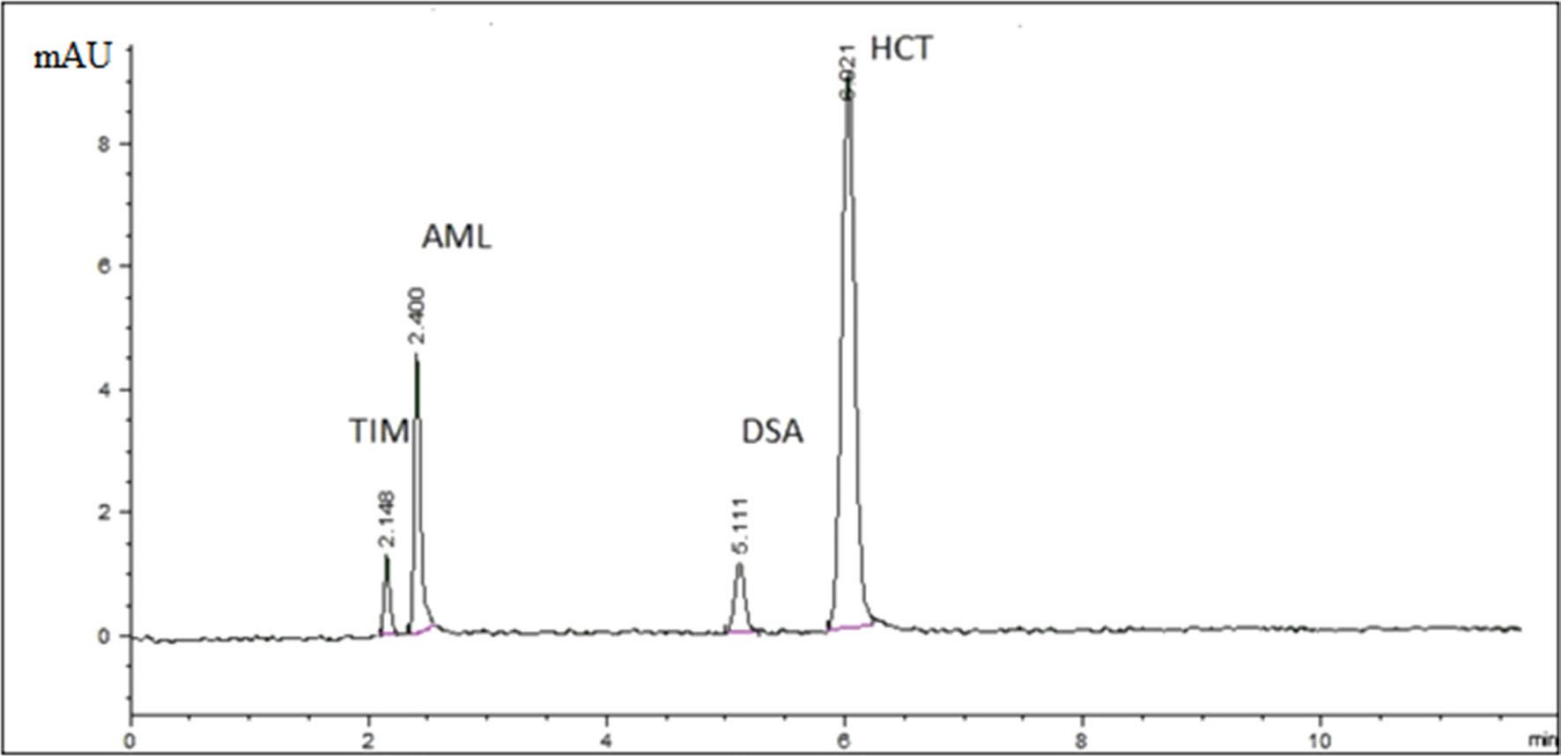
CZE-DAD electropherogram showing separation of mixture of AML (30.0 µg/mL), TIM (30.0 µg/mL), HCT (50.0 µg/mL), and DSA (10.0 µg/mL) using an uncoated fused-silica capillary with a total length of 48.5 cm and an effective length of 40 cm (50 μm i.d.); UV detection at 200.0 nm; sample injection: 50 mbar for 5 s; an applied voltage of + 15.0 kV; and a BGE of borate buffer (pH 9.0; 40.0 mM)

In CZE, buffer type and concentration, pH and applied voltage play a chef role in electrophoretic separation optimization. Two buffers were tested as background electrolyte, namely; borate and phosphate buffers. Poor electrophoretic separation of cited compounds was achieved using phosphate buffer. Therefore, borate buffer was chosen as BGE. Different pH values were tried taking in consideration the physico-chemical properties of studied analytes. It was observed that upon using acidic pH, poor separation was obtained between AML, HCT and TIM. Upon increasing pH to more basic values separation was greatly enhanced. Different borate buffer concentrations were tried under constant instrumental conditions. Finally, it was found that 40.0 mM borate buffer of pH 9.0 give better peak shapes with good resolution. Upon optimization of the applied voltage, applying voltage of + 20 kV resulted in increased current value and peaks deterioration, while upon decreasing voltage value to + 12 kV, increased run time was observed. Applying voltage of + 15 kV gave better separation with short run time. The dilution solvent used to prepare the samples was another significant factor that was investigated in order to improve the method’s sensitivity. Despite adjusting the pH, buffer salt, strength, and applied voltage, a problem of the co-elution of TIM and AML with ethanol (if used as a dilution solvent) was encountered with a decrease in sensitivity as well as a worsening of the peak shapes. Even though CT could be better solubilized and well resolved, Additional file 1: Fig. S1. As a result, the procedure was shifted to use water as a diluent in order to alleviate this issue, an improved peak shape of AML and TIM with better resolution was observed. Unfortunately, the CT peak abruptly vanished, demonstrating the need for an organic solvent for CT sensitive measurement. Accordingly, CT was excluded from further practical studies. Therefore, water was chosen as a solvent in order to efficiently determine the major compounds; AML, TIM, and HCT as well as DSA, Fig. 3. Finally, different detection wavelengths were investigated including 200.0 nm, 210.0 nm, 225.0 nm, 240.0 nm and 260.0 nm and it was found that best sensitivity was achieved at 200.0 nm.

### Assessment of the proposed methods’ greenness

Recently, many assessment tools were developed to evaluate the environmental impact of analytical method [25–28]. One of the simplest and oldest qualitative assessment tools is NEMI. It is represented as circle divided into four fields, each field represents different aspect of the analytical method [3]. It evaluates the analytical methodology in four key terms reflected in the four fields of the circle as follow: (1) PBT (Persistent, bio-accumulative and toxicity) of the chemicals used, (2) hazards of the used solvents, (3) corrosiveness and (4) the amount of generated wastes. The field is shaded green if it met the greenness requirements. The suggested CZE-DAD method is said to be a greener one since it exhibits the fortunately prevalent green color of the four fields, as none of the used solvents neither appears on the PBT list nor in the Environmental Protection Agency (EPA) Toxic Release Inventory (TRI) chemicals list [29] and the pH of the used systems lies in the acceptable range (between 2 and 12). Additionally, the amount of the generated wastes is less than 50 g per analyzed sample, Table 1. On the other hand, the proposed HPTLC-densitometric method displays 3 of the 4 fields as green shaded, indicating the use of hazardous solvents in the developing system, Table 1. Although, NEMI is considered a simple, easy to read assessment tool, it only provides qualitative information about the proposed method greenness besides ignoring some important aspects in analytical process like energy consumption and health hazards.

**Table 1:** Greenness assessment of the two proposed separation methods by analytical eco-scale, NEMI, GAPI and AGREE tools

For HPTLC-densitometric method				
Eco-scale assessment		NEMI pictogram	GAPI assessment	AGREE assessment
Reagents	Penalty points (PPs)			
Ammonia (25%)	6			
Water	0			
Ethanol	4			
Ethyl acetate	4			
Instrument				
Energy consumption	1			
Occupational hazard	3			
Waste	6			
Total PPs	24			
Analytical eco-scale total score	76			
Comment	Excellent green analysis			

For CZE-DAD method				
Eco-scale assessment		NEMI pictogram	GAPI assessment	AGREE assessment
Reagents	Penalty points (PPs)			
Sodium tetraborate decahydrate	4			
Water	0			
Instrument				
Energy consumption	2			
Occupational hazard	0			
Waste	4			
Total PPs	10			
Analytical eco-scale total score	90			
Comment	Excellent green analysis			

Analytical Eco-scale system is a comprehensive semi-quantitative system that assigns penalty scores to each parameter, including the quantity and danger of the employed reagents, waste production, energy use, different waste treatment methods, and any potential occupational concerns, in the developed method that is not in agreement with the ideal green analysis [30]. Then, subtracting the total penalty score from a base of 100 (that is assigned for the ideal green method). An unsatisfactory green analysis is one that receives a score of less than 50 whereas an exceptional green analysis receives a score of greater than 75. The eco-scale scores displayed in Table 1 indicate that the two developed methods follow the green analytical chemistry concepts (GAC) and has an excellent green practice. The obtained scores reinforced the NEMI findings, ranking the proposed CZE-DAD method above the HPTLC-densitometric one with a higher score of 90 and the HPTLC-densitometric method with a lower score of 76, Table 1. Despite the fact that eco-scale system takes into consideration more experimental parameters than NEMI, it is still considered as semi-quantitative tool as the final score lacks sufficient information about the nature of hazards and thereby can’t be used to identify the analytical method’s drawbacks.

Additionally, Green Analytical Procedure Index (GAPI) is a recently designed tool that evaluate the greenness of the entire analytical method starting initially from sample preparation and collection till the final step of determination [31]. It is represented by a pictogram consisting of five pentagrams using three different color codes: green, yellow and red which can be translated to low, medium and elevated environmental influence of each parameter. The developed CZE-DAD method possess higher green shaded sections (5) and less red shaded ones (2) compared to the developed HPTLC-densitometric method which shows (4) green shaded sections and (3) red shaded ones, Table 1. GAPI tool provides a full evaluation of the analytical methodology, giving detailed information about every aspect of the analytical method and thus, facilitate identifying areas that still need to be improved in terms of greenness. Even though the primary disadvantage of the GAPI tool is that it is more complicated than NEMI and eco-scale ones.

AGREE is a downloadable software newly introduced to assess the environmental impact of the developed analytical method taking in consideration the 12 principle of the GAC [32]. The result is transferred into a pictogram with 12 sector and a final score appear in the middle scaling from 0 to 1. Each sector of the pictogram is colored using a color scale system from green to red according to the environmental impact. The best approach yields a score of 1, using the color dark green. The developed methodologies' AGREE pictograms in Table 1 demonstrate their greenness with an overall score of 0.63 and 0.61, for the proposed HPTLC-densitometry and CZE-DAD methods, in order. As a result, AGREE metric is regarded as being user-friendly, thorough, simple to use, and extremely quick.

Briefly, the usefulness of the proposed separation-based approaches depends on their capacity to reduce the usage and consumption of the solvents and instrumental energy. Since they may be used safely and without endangering the environment or the analyst, the suggested procedures are referred to as “green approaches.” Additionally, the benefit of small volumes of aqueous buffers usage in CZE-DAD is adequately demonstrated by the high score attained by the analytical eco-scale as well as by the predominance of green color in both NEMI and GAPI pictograms. Although the CZE-DAD method is thought to be more environmentally friendly, it has certain drawbacks, such as the requirement for expensive and sophisticated instrumentation and well-trained operator. In addition, CZE-DAD method failed to simultaneously determine CT with the other four compounds. On the other hand, HPTLC-densitometry is believed to be more straightforward with high sample throughput and independent on costly instrumentation or solvents with the unique capability of the determination of the cited drugs along with HCT impurities, CT and DSA.

### Validation of the proposed methods

Validation of the developed methods was conducted according to the ICH guidelines [33] using the finally optimized experimental conditions, Table 2.

**Table 2:** Regression and validation parameters of the proposed HPTLC-densitometry and CZE-DAD method

Method parameter	HPTLC-densitometric method					CZE-DAD method			
	AML	HCT	TIM	DSA	CT	AML	HCT	TIM	DSA
Range (µg/band for HPTLC, and µg/mL for CZE)	0.5–10.0	1.0–16.0	1.0–14.0	0.05–1.0	0.05–1.0	20.0–160.0	10.0–200.0	10.0–120.0	10.0–100.0
Slope (b)^a^	2160.7	–	2303.3	–	–	0.1696	0.1993	0.0923	0.2814
Coefficient 1 (b1)^b^	–	− 85.058	–	− 11,534	− 1790.9	–	–	–	–
Coefficient 2 (b2)^b^	–	3363.9	–	25,358	6175.2	–	–	–	–
Intercept (a)^a, b^	1793.8	10,404	5197	− 478.24	161.03	1.8958	3.2617	0.6533	0.5594
Correlation Coefficient (r)	0.9999	0.9998	0.9998	0.9998	0.9998	0.9997	0.9998	0.9997	0.9996
Accuracy (Mean ± SD)	100.81 ± 0.89	99.90 ± 1.59	100.10 ± 1.39	99.44 ± 1.36	100.53 ± 1.02	100.16 ± 1.65	99.67 ± 1.13	99.99 ± 1.75	100.02 ± 1.21
*Precision*									
(± %RSD)^c^	0.80	0.47	0.68	0.64	0.30	0.88	1.29	0.62	0.99
(± %RSD)^d^	1.01	1.44	0.81	1.63	1.03	1.10	1.64	1.43	1.74
LOD^e^ (µg/band for HPTLC, and µg/mL for CZE)	–	–	–	0.01	0.01	–	–	–	2.94
LOQ^e^ (µg/band for HPTLC, and µg/mL for CZE)	–	–	–	0.03	0.02	–	–	–	8.92
Robustness^f^	1.21	1.43	1.11	1.31	0.98	1.76	1.45	1.78	1.53

^a^Regression equation for CZE-DAD: *A* = *a* + *bc*, where ‘A’ is the average corrected peak area and ‘c’ is the concentration (μg/mL). While for HPTLC-densitometry: *A* = *a* + *bc*, where ‘*A*’ is the average peak area and ‘c’ is the concentration (μg/band)

^b^Coefficient 1 and 2 are the coefficients of *x*^2^ and *x*, respectively. Following a polynomial regression: *A* = *b*1*x*^2^ + *b*2*x* + *a*, where ‘A’ is the average peak area, ‘c’ is the concentration (μg/band), ‘b1’ and ‘b2’ are coefficients 1 and 2, respectively and ‘a’ is the intercept

^c^Intra-day precision [average of three different concentration of three replicates each (n = 9) within the same day], for HPTLC the concentrations were (2.0, 4.0, 6.0 µg/band) for AML, (4.0, 6.0, 8.0 µg/band) for TIM, (8.0, 10.0, 14.0 µg/band) for HCT and (0.2, 0.5, 1.0 µg/band) for DSA and CT. For CZE: the concentrations were: (80.0, 100.0, 120.0 µg/mL) for AML, (80.0, 100.0, 120.0 µg/mL) for TIM, (140.0, 160.0, 180.0 µg/mL) for HCT and (40.0, 60.0, 100.0 µg/mL) for DSA and CT

^d^Inter-day precision [average of three different concentration of three replicates each (n = 9) repeated on three successive days], the concentrations were the same as in intra-day precision

^e^LOD and LOQ are calculated according to ICH, 3.3 × SD of the residuals/slope and 10 × SD of the residuals/slope, respectively

^f^For HPTLC−densitometry: average of the change in wavelength (±1 nm) and saturation time (± 5 min). For CZE−DAD: average of the change in applied voltage (± 1 kV)

#### Linearity

Linearity of the proposed methods was estimated by plotting calibration curves relating the peak area or corrected peak area to the corresponding concentration of each component and regression parameters were computed Table 2.

#### Limits of detection (LOD) and limits of quantification (LOQ)

Limits of detection (LOD) and limits of quantification (LOQ) of HCT officially specified impurities were evaluated in accordance to the ICH guidelines using the standard deviation of residuals and slope.

#### Selectivity

The selectivity of the proposed methods is guaranteed by the successful resolution of the studied compounds as shown in Figs. 2 and 3. Additionally, the good percentage recoveries obtained for the analysis of AML, HCT and TIM in their co-formulated tablets suggest the absence of any interference from the common tablet excipients, Table 4 and Additional file 1: Fig. S2.

Peak identity and purity were confirmed using the winCATS spectral correlation tool for HPTLC-densitometric method and online by DAD for CZE-DAD method. The purity index of the studied analytes did not surpass the threshold limits in any of the analyzed samples along with the superimposed UV spectra, indicating the homogeneity of the separated peaks.

#### Accuracy

For the estimation of the developed methods accuracy, a minimum of five different pure samples of each component covering the specified calibration range were analyzed in triplicates. The developed methods are found to exhibit acceptable accuracy based on the calculated percentage recoveries, Table 2.

#### Precision

To evaluate method precision, three discrete levels of concentration of each component were analyzed in triplicates in the same day and on three consecutive days. The obtained results indicate good method precision as reinforced by RSD% values less than 2, Table 2.

#### Robustness

Reliability of the developed method was checked by inducing minor changes in the separation conditions and then monitoring method performance. For HPTLC-densitometric method, changing the scanning wavelength (± 1 nm) or saturation time of the development chamber (± 5 min). For CZE-DAD method, changing the applied voltage (15 ± 1 kV). The low % RSD values obtained indicate robustness of the proposed methods, Table 2.

#### System suitability parameters

Parameters of system suitability were then checked [13, 34], to assure the developed methods’ performance and the results were summarized in Table 3.

**Table 3:** System suitability parameters of the proposed HPTLC-densitomety and CZE-DAD methods

Method	Parameter	CT	AML	TIM	HCT	DSA	Reference value [34]
HPTLC-densitometry	Retardation factor (*R*_*f*_) ± 0.02^a^	0.18	0.28	0.44	0.64	0.74	
	Capacity factor (*k*′)^b^	4.56	2.57	1.27	0.56	0.35	
	Selectivity (α)^c^	1.77	2.02	2.27		1.60	> 1
	Resolution (R_s_)^d^	1.89	2.73	2.81		1.76	R_s_ > 1.5
	Tailing factor (T)	0.99	1.00	0.92	1.01	1.12	T ≤ 2
	Number of theoretical plates (N)	246.49	317.48	825.25	855.5	3816.49	

	Parameter	TIM	AML	DSA	HCT	Reference value [13]
CZE-DAD	Selectivity (α)^e^	1.12	1.94		1.18	> 1
	Resolution (R_s_)^e^		2.00	24.00	5.70	Rs > 1.5
	Tailing factor (T)	1.15	1.12	1.03	1.12	T ≤ 2
	Number of theoretical plates (N)^f^	9216	4993.78	51,076	11,320.96	N ˃ 2000
	Number of theoretical plates per meter (TPM)^g^	19,002.06	10,296.44	105,311.34	23,342.18	
	Migration time (min ± 0.2)	2.15	2.40	5.11	6.02	

^a^Retardation factor (*R*_*f*_) = distance travelled by the analyte/distance travelled by the solvent front

^b^Capacity factor (*k*′) = (1 − *R*_*f*_)/*R*_f_

^c^Selectivity (α) = *k*′_2_/*k*′_1_ calculated for each of two successive peaks

^d^Resolution (R_s_) = *z*_2_ − *z*_1_/0.5 (w_1_ + w_2_), where *z*_2_ − *z*_1_ is the distance between two adjacent spot centers and w is the peak width calculated for each of two successive peaks

^e^Selectivity and resolution as extracted from the software

^f^Number of theoretical plates (N) = 16(T_R_/W)^2^

^g^Number of theoretical plates per meter (TPM) = [1600(T_R_/W)^2^]/L, where L is the total capillary length in cm

### Assay of pharmaceutical formulation

Using a straightforward extraction approach, the improved and validated procedures were successively employed for the study of the specified drugs combination in their commercially available pharmaceutical formulation, Table 4. Good results with minimal sample preparation were obtained, ensuring the reliability of the proposed methods for the determination of cited drugs in the presence of from the common tablets’ excipients without any conflict. Additionally, standard addition technique was performed and the proposed methods’ validity was further verified, Table 4.

**Table 4:** Results obtained by applying the proposed HPTLC-densitometry method and CZE method for the determination of amiloride hydrochloride, timolol maleate and hydrochlorothiazide in moducren^®^ tablets and application of standard addition technique

Pharmaceutical formulation	HPTLC-densitometry					CZE-DAD method			
	Drug	%Found ± SD^a^	Standard addition technique			%Found ± SD^a^	Standard addition technique		
			Claimed (µg/band)	Pure added (µg/band)	%R of the pure added^b^		Claimed (μg/mL)	Pure added (μg/mL)	%R of the pure added amount^b^
Moducren^®^ tablets B.N. AJC 066 (each tablet is labelled to contain 2.5 mg AML, 10 mg TIM and 25 mg HCT)	**AML**	97.96 ± 1.25	1.0	0.5	97.20	98.34 ± 1.10	40.0	20.0	98.95
				1.0	98.61			40.0	100.17
				2.0	99.98			80.0	100.22
					**Mean ± SD**	98.59 ± 1.39		**Mean ± SD**	99.78 ± 0.72
	**HCT**	99.76 ± 0.99	5.0	2.5	97.53	100.47 ± 1.18	50.0	25.0	100.69
				5.0	100.29			50.0	101.11
				10.0	99.78			100.0	99.26
					**Mean ± SD**	99.20 ± 1.47		**Mean ± SD**	100.35 ± 0.97
	**TIM**	100.77 ± 1.10	4.0	2.0	100.43	99.26 ± 1.02	40.0	20.0	100.13
				4.0	99.52			40.0	98.40
				8.0	101.43			80.0	**99.79**
					**Mean ± SD**	99.20 ± 1.47		**Mean ± SD**	99.44 ± 0.92

^a^Average of five determinations

^b^Average of three determinations

Additionally, the proposed methods are statistically compared to the different official ones for the determination of the cited drugs in their pure forms [13, 16]. As shown in Additional file 1: Table S1, the calculated student’s t and F-test demonstrate that no significant differences were obtained in terms of both precision and accuracy.

## Conclusion

A fixed dose combination of amiloride hydrochloride, hydrochlorothiazide and timolol maleate is presented as a new therapy for hypertension. In the current work, sustainable, reliable and robust separation methods were developed for quantitative estimation of AML, HCT and TIM together with HCT related impurities, CT and DSA, presenting the first HPTLC-densitometry and capillary zone electrophoresis methods to be reported for simultaneous determination of this combination therapy. The usage of harmless and less dangerous solvents was carefully considered. The developed methods were successively applied for the determination and purity assessment of the cited drugs in Moducren^®^ Tablets. The greenness of the proposed methods was comprehensively evaluated using recently introduced assessment tools. Clearly, the established methods display excellent analytical performance with low environmental impact which encourage their use for routine analysis of the cited drugs either in their pure powder form or in pharmaceutical formulation in quality control laboratories.

## Supplementary Information

**Additional file 1:Table S1**. Statistical comparison of the results obtained by the proposed HPTLC-densitometry and CZE-DAD method and those of the official methods for the analysis of pure AML, HCT and TIM. **Figure S1**. CZE-DAD electropherogram showing separation of mixture of AML (30.0 μg/mL), TIM (30.0 μg/mL), HCT (50.0 μg/mL), DSA (10.0 μg /mL) and CT (10.0 μg /mL) using ethanol as a diluent and an uncoated fused-silica capillary with a total length of 48.5 cm and an effective length of 40 cm (50 μm i.d); UV detection at 200.0 nm; sample injection: 50 mbar for 5 s; an applied voltage of +15.0 kV; and a BGE of borate buffer (pH 9.0; 40.0 mM). **Figure S2**: (a) HPTLC-densitogram of Moducren^®^ Tablets extract, using ethyl acetate-ethanol-water-ammonia (8.5:1.0:0.5:0.3, by volume) as a developing system at 220.0 nm. (b) CZE-DAD electropherogram of Moducren^®^ Tablets extract, using an uncoated fused-silica capillary with a total length of 48.5 cm and an effective length of 40 cm (50 μm i.d); UV detection at 200.0 nm; sample injection: 50 mbar for 5 s; an applied voltage of +15.0 kV; and a BGE of borate buffer (pH 9.0; 40.0 mM).

## Data Availability

All datasets used and/or analysed during the current study available from the corresponding author on reasonable request.

## Abbreviations

AML: Amiloride hydrochloride
HCT: Hydrochlorothiazide
TIM: Timolo Maleate
DSA: Salamide
CT: Chlorothiazide
CZE: Capillary Zone Electrophoresis
ICH: International Council of Harmonization
NEMI: National Environmental Method Index
GAPI: Green Analytical Procedure Index
TRI: Toxic Release Inventory
GAC: Green Analytical Chemistry
LOD: Limit of Detection
LOQ: Limit of Quantification
