# Supplementary material for: An eco-friendly separation-based framework for quantitative determination and purity testing of an antihypertensive ternary pharmaceutical formulation
Source: BMC Chem. 2023 Mar 10;17(1):14. doi: 10.1186/s13065-023-00926-1 (PMC10007836; doi:10.1186/s13065-023-00926-1)
Supplement: Supplementary file 1 — Additional file 1:Table S1. Statistical comparison of the results obtained by the proposed HPTLC-densitometry and CZE-DAD method and those of the official methods for the analysis of pure AML, HCT and TIM. Figure S1. CZE-DAD electropherogram showing separation of mixture of AML (30.0 μg/mL), TIM (30.0 μg/mL), HCT (50.0 μg/mL), DSA (10.0 μg /mL) and CT (10.0 μg /mL) using ethanol as a diluent and an uncoated fused-silica capillary with a total length of 48.5 cm and an effective length of 40 cm (50 μm i.d); UV detection at 200.0 nm; sample injection: 50 mbar for 5 s; an applied voltage of +15.0 kV; and a BGE of borate buffer (pH 9.0; 40.0 mM). Figure S2: (a) HPTLC-densitogram of Moducren® Tablets extract, using ethyl acetate-ethanol-water-ammonia (8.5:1.0:0.5:0.3, by volume) as a developing system at 220.0 nm. (b) CZE-DAD electropherogram of Moducren® Tablets extract, using an uncoated fused-silica capillary with a total length of 48.5 cm and an effective length of 40 cm (50 μm i.d); UV detection at 200.0 nm; sample injection: 50 mbar for 5 s; an applied voltage of +15.0 kV; and a BGE of borate buffer (pH 9.0; 40.0 mM). [file 13065_2023_926_MOESM1_ESM.docx]

**Table S1.** Statistical comparison of the results obtained by the proposed HPTLC-densitometry and CZE-DAD method and those of the official methods for the analysis of pure AML, HCT and TIM.

| **Parameter** | **HPTLC-densitometry** | | | **CZE-DAD method** | | | **Official method** | | |
| --- | --- | --- | --- | --- | --- | --- | --- | --- | --- |
|  | **AML** | **HCT** | **TIM** | **AML** | **HCT** | **TIM** | **AML (13)** | **HCT (13)** | **TIM (16)** |
| **Mean** | 100.82 | 99.17 | 100.71 | 100.35 | 99.98 | 99.25 | 99.81 | 100.24 | 99.93 |
| **SD** | 0.80 | 1.31 | 0.87 | 0.88 | 1.28 | 1.41 | 1.51 | 1.42 | 1.12 |
| **Variance** | 0.64 | 1.71 | 0.76 | 0.77 | 1.64 | 1.99 | 2.28 | 2.02 | 1.25 |
| **n** | 5 | 5 | 5 | 5 | 5 | 5 | 5 | 5 | 5 |
| **Student’s *t*-test**  (2.306)* | 1.321 | 1.238 | 1.230 | 0.691 | 0.371 | 0.845 | --- | --- | --- |
| ***F-*test**  (6.39)* | 3.56 | 1.18 | 1.65 | 2.95 | 1.23 | 1.59 | --- | --- | --- |

* Figures between parentheses represent the corresponding tabulated values of t and F at P = 0.05.


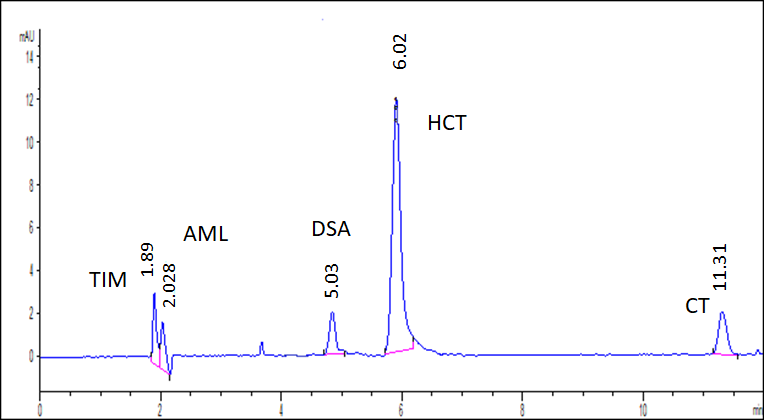


**Figure S1.** CZE-DAD electropherogram showing separation of mixture of AML (30.0 µg/mL), TIM (30.0 µg/mL), HCT (50.0 µg/mL), DSA (10.0 µg /mL) and CT (10.0 µg /mL) using ethanol as a diluent and an uncoated fused-silica capillary with a total length of 48.5 cm and an effective length of 40 cm (50 μm i.d); UV detection at 200.0 nm; sample injection: 50 mbar for 5 s; an applied voltage of +15.0 kV; and a BGE of borate buffer (pH 9.0; 40.0 mM).


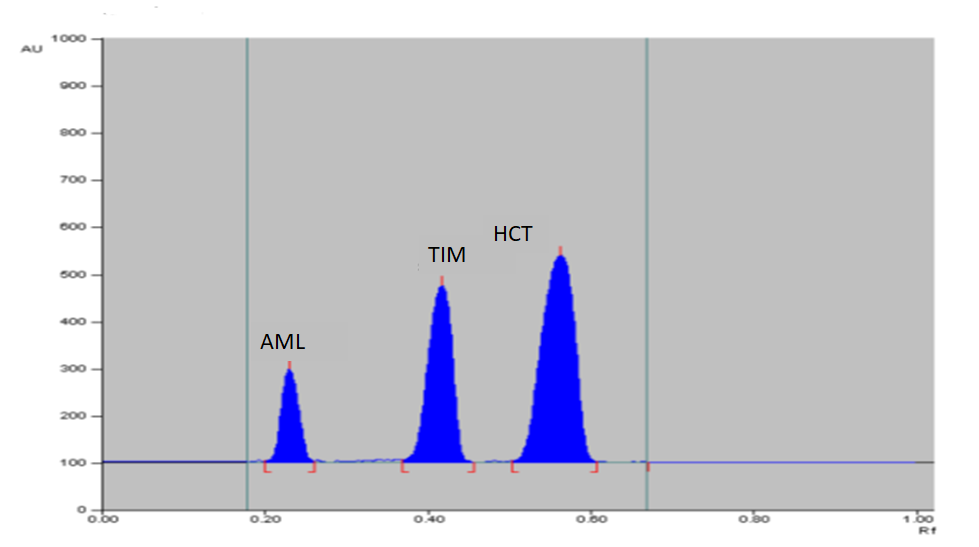


(a)


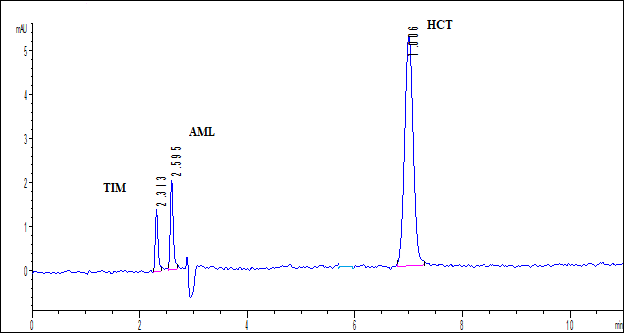


(b)

**Figure S2:** (a) HPTLC-densitogram of Moducern^®^ Tablets extract, using ethyl acetate-ethanol-water-ammonia (8.5:1.0:0.5:0.3, by volume) as a developing system at 220.0 nm. (b) CZE-DAD electropherogram of Moducern^®^ Tablets extract, using an uncoated fused-silica capillary with a total length of 48.5 cm and an effective length of 40 cm (50 μm i.d); UV detection at 200.0 nm; sample injection: 50 mbar for 5 s; an applied voltage of +15.0 kV; and a BGE of borate buffer (pH 9.0; 40.0 mM).
